# Supplementary material for: X-ray structure of LeuT in an inward-facing occluded conformation reveals mechanism of substrate release
Source: Nat Commun. 2020 Feb 21;11:1005. doi: 10.1038/s41467-020-14735-w (PMC7035281; doi:10.1038/s41467-020-14735-w)
Supplement: Supplementary file 1 — Supplementary Information [file 41467_2020_14735_MOESM1_ESM.docx]

**SUPPLEMENTARY INFORMATION**

X-ray structure of LeuT in an inward-facing occluded conformation reveals mechanism of substrate release

Gotfryd et. al

**Supplementary Figure 1.**

**
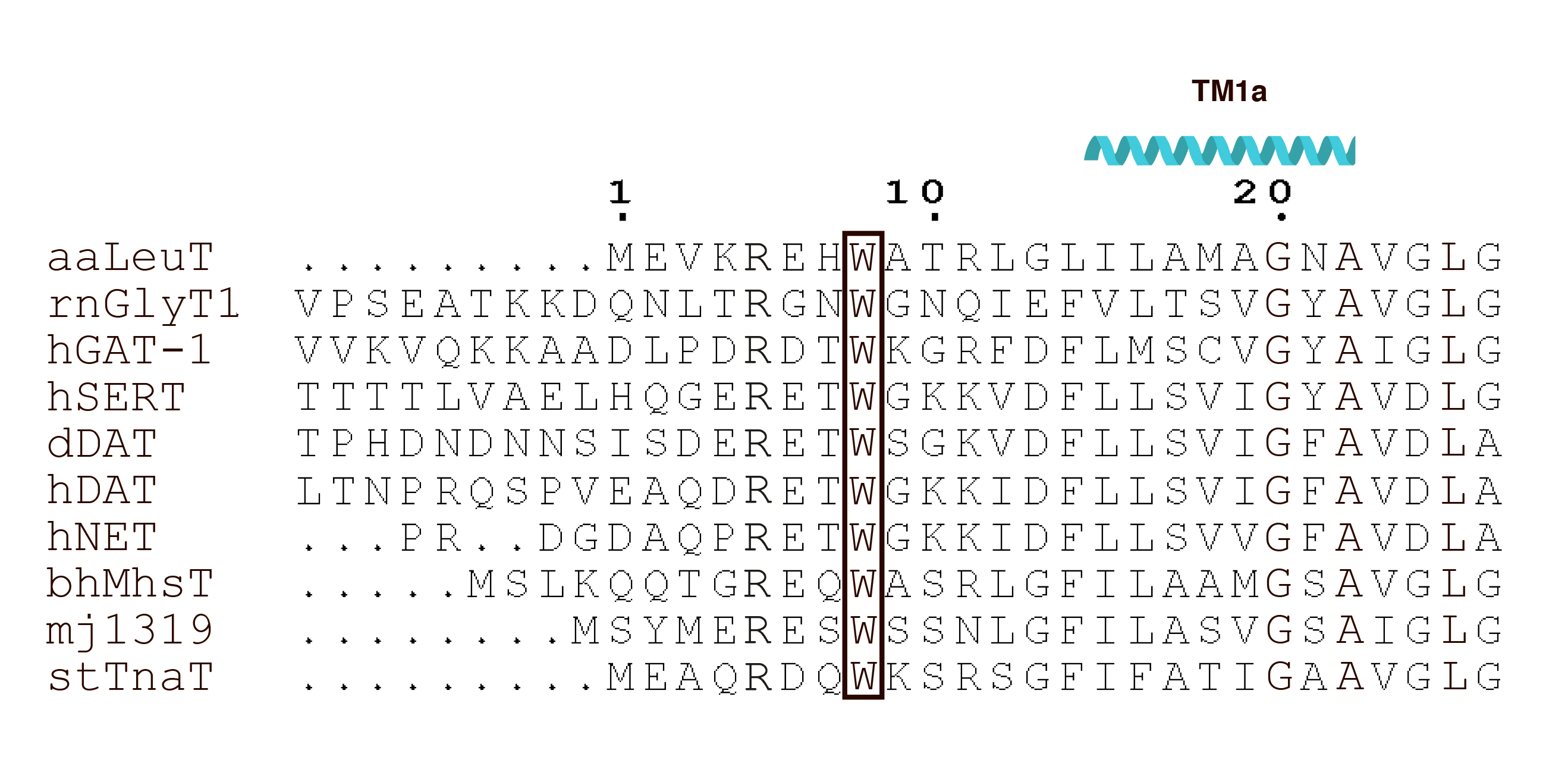
**

## **Supplementary Figure 1. Primary sequence of the N-terminus of selected NSS members.** Sequence alignment of the N-terminal segment of Aquifex aeolicus LeuT (aaLeuT; O67854), Rattus norvegicus GlyT1 (rnGlyT1; P28572), Homo sapiens GAT-1 (hGAT-1; P30531), Homo sapiens SERT (hSERT; P31645), Drosophila melanogaster DAT (dDAT; Q7K4Y6), Homo sapiens DAT (hDAT; Q01959), Homo sapiens NET (hNET; P23975), Bacillus halodurans MhsT (bhMhsT; Q9KDT3), Methanocaldococcus jannaschii 1319 (mj1319; Q58715) and Symbiobacterium thermophilum TnaT (stTnaT; O50649). Abbreviated names and UniProt accession numbers of full-length NSS protein sequences are shown in brackets. Sequences were aligned using Clustal Omega (https://www.ebi.ac.uk/Tools/msa/clustalo/) and edited in ESPript 3 (http://espript.ibcp.fr/ESPript/ESPript/). The conserved W8 residue of LeuT is framed and the position of transmembrane segment (TM) 1a is indicated.

**Supplementary Figure 2.**

**Supplementary Figure 2. Conformational dynamics of the intracellular gates in LeuT_W8A_ and LeuT_WT_.** Histograms for frequencies of the R5-D369 or Y268-Q361 distances and of the χ1 dihedral angle of the Y268 residue obtained from analysis of the ensemble MD simulations of the W8A_OUT-OCC_ (**a-c**, top panel) and WT_OUT-OCC_ (**d-f**, bottom panel) LeuT constructs. The data for each of the 10 individual replicates are shown in different colors.

**Supplementary Figure 3.**

**
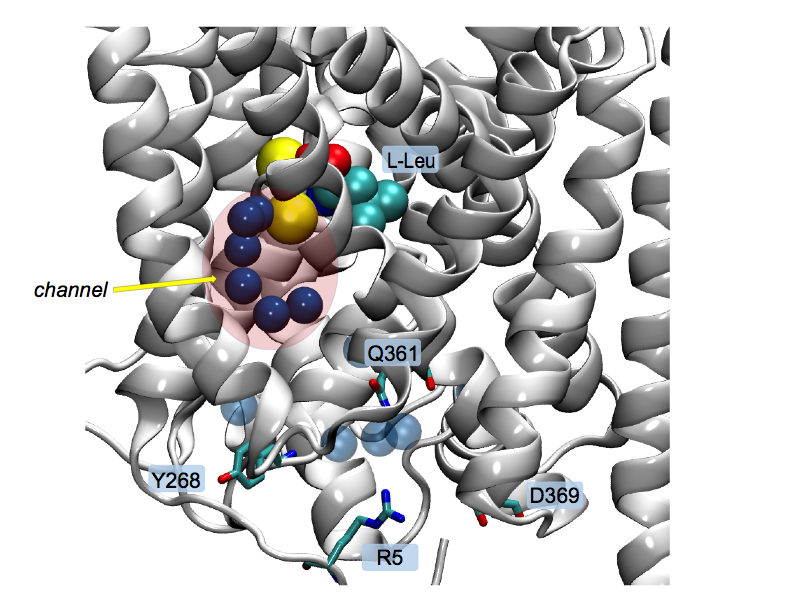

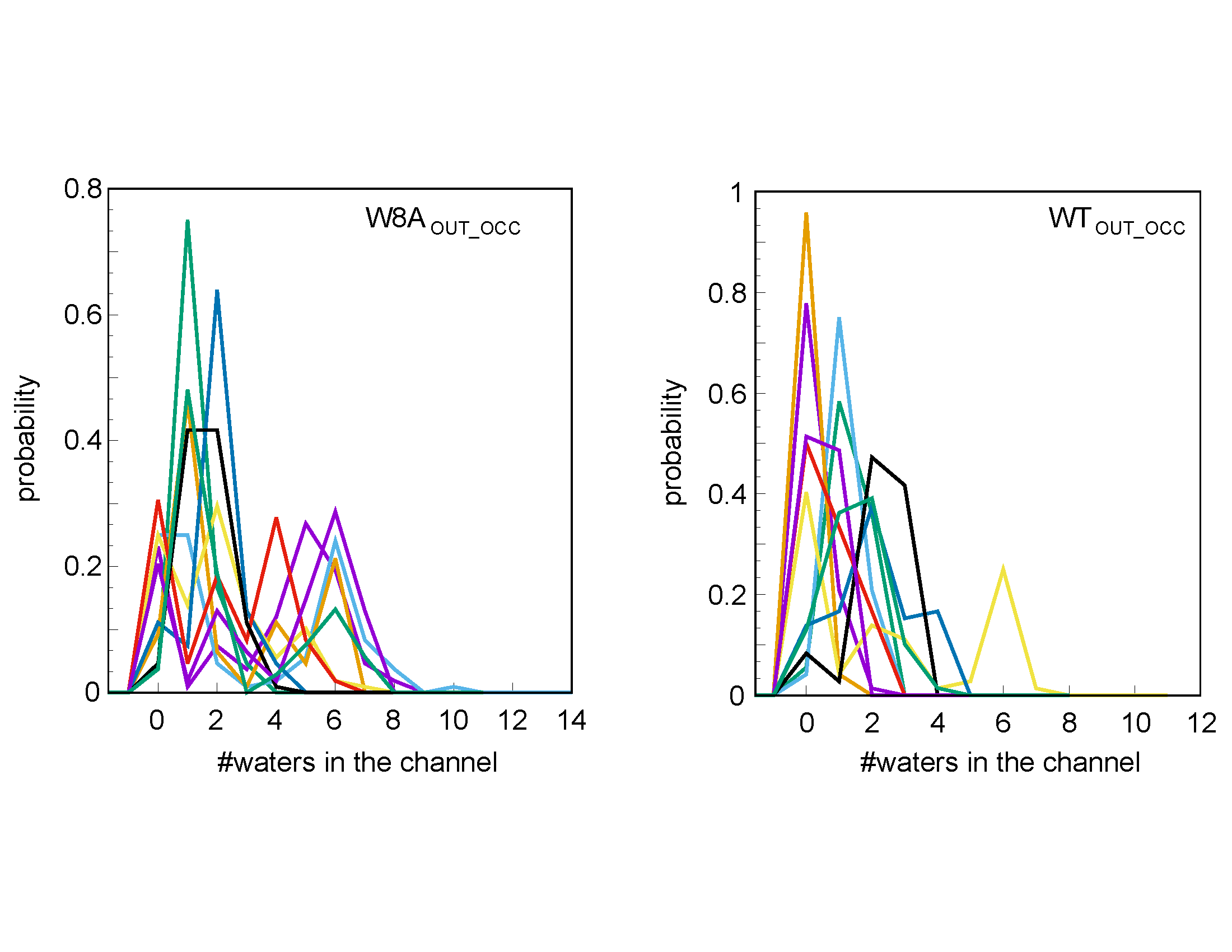

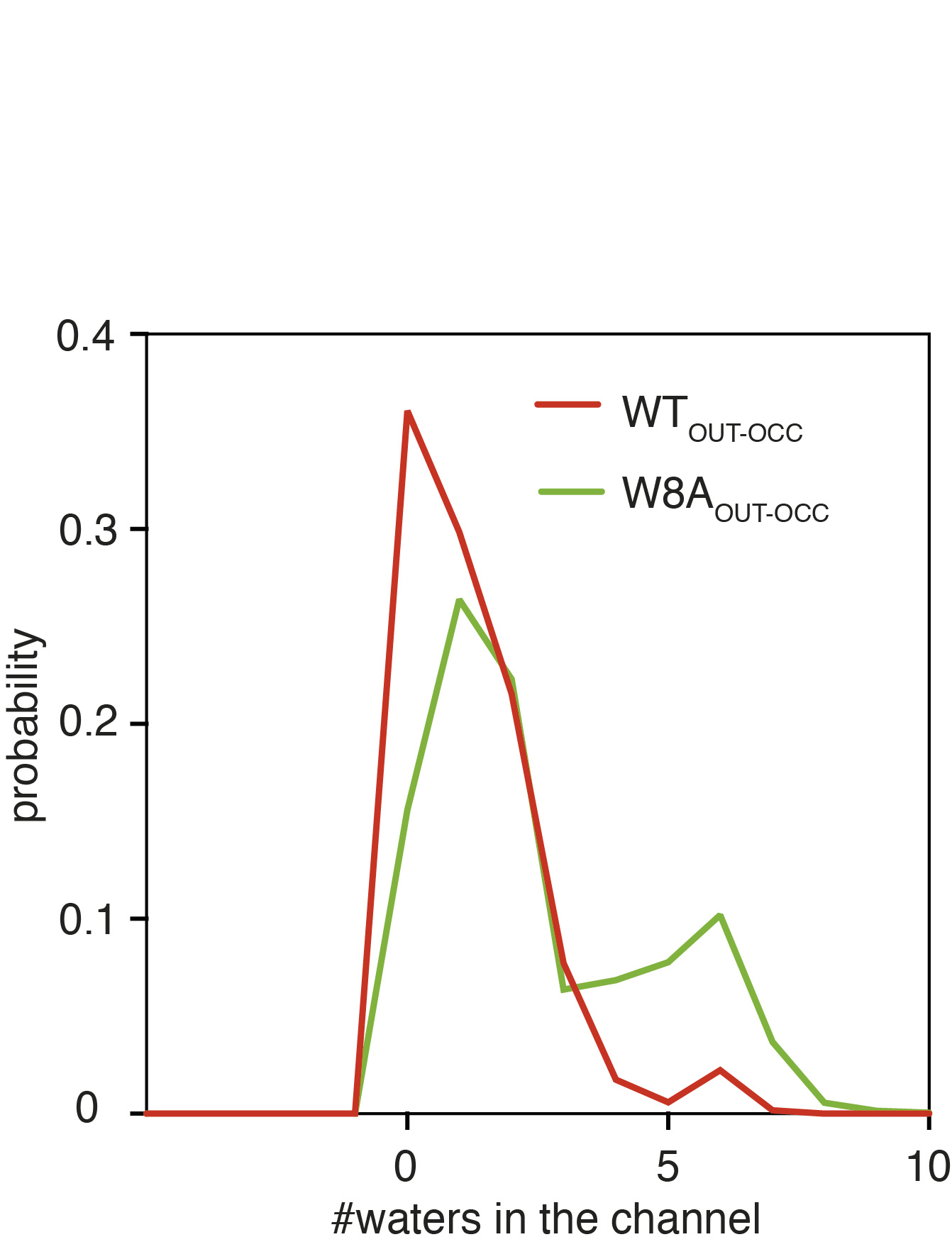
­­**

b

a

d

c

**Supplementary Figure 3. Water penetration to the Na2 site sodium ion in LeuT_WT_ and LeuT_W8A_. a** Histograms of the water counts in the channel region of the intracellular vestibule obtained from analysis of the ensemble MD simulations of WT_OUT-OCC_ (red line) and W8A_OUT-OCC_ (green line) LeuT constructs. **b** Structural representation of the W8A_OUT-OCC_ LeuT highlighting the location of the intracellular channel (*semi-transparent red circle*). The protein is shown in white cartoon, the residues constituting the internal gate (R5, D369, Y268 and Q361) in licorice and labeled, the two bound Na^+^ ions in yellow spheres and labeled, bound substrate (L-Leu) in space fill and labeled and oxygen atoms of the water molecules in the intracellular vestibule are depicted as blue spheres (the waters in the channel and outside it are shown in opaque and transparent representations, respectively). **c-d** Histogram of number of waters in the channel from the analysis of the 10 individual trajectories of the W8A_OUT-OCC_ (**c**) and WT_OUT-OCC_ (**d**) systems. Water wires connecting the Na2 site to the intracellular vestibule are formed in half of the W8A_OUT-OCC_ trajectories, but only in one trajectory for the WT_OUT-OCC_ system (see probabilities for number of water molecules ≥6).

## **Supplementary Figure 4.**

**
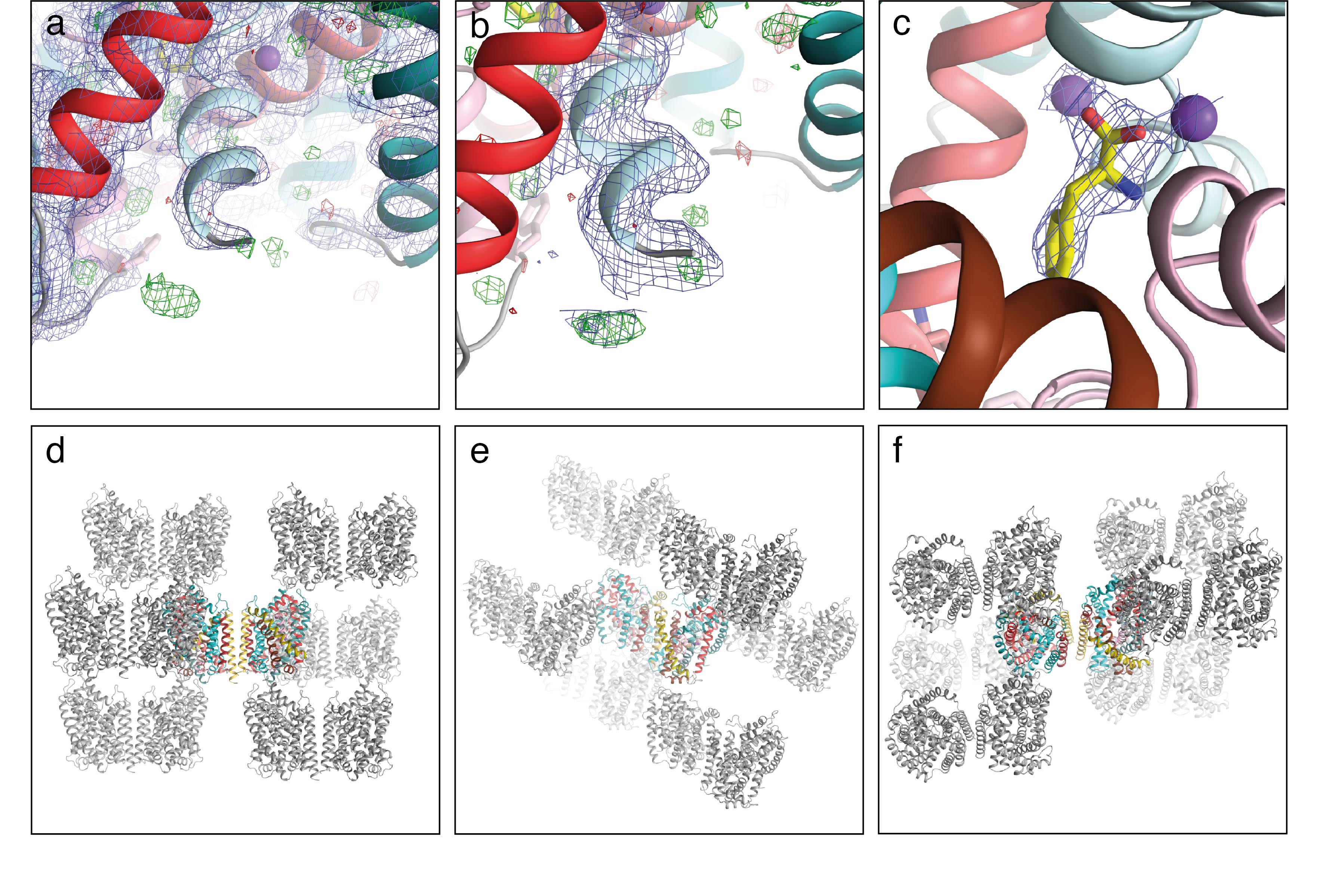
­­**

## **Supplementary Figure 4. Electron densities of the N-terminal region, omit maps of sodium ions and substrate, and crystal packing of LeuT_W8A_.** **a** Close view of the N-terminal region with the final 2F_O_-F_C_ (purple, σ=1.0) and F_O_-F_C_ (green and red mesh for positive and negative densities, respectively, σ=3.0) electron densities. **b** Close view of the N-terminal region with the final 2F_O_-F_C_ (purple, σ=0.5) and F_O_-F_C_ (green and red mesh for positive and negative densities, respectively, σ=3.0) electron densities. Coloring is identical as in Fig. 2. No visible electron density is found for the first 12 N-terminal residues, suggesting that the N-terminus is disordered in the crystals. **c** Omit electron density maps of bound sodium ions and substrate. Fo-Fc electron densities (purple) contoured to 1.0 σ showing the evidence for Na1- and 2-bound sodium ions and L-Phe. **d-f** Crystal packing of LeuT_W8A_ in the P1 crystal form shown along the three axes, respectively. Colored are single LeuT_W8A_ molecules where no crystal contacts are observed close to the intracellular parts of TM1a and TM5. The N-terminus is unperturbed by the crystal packing.

**Supplementary Figure 5.**

**­­­**

**Supplementary Figure 5.** **Comparison of the sodium and substrate binding sites in LeuT_W8A_ (light blue) and the LeuT outward-facing occluded state (PDB-ID: 2A65) (light pink).** Left panel shows Na1 (blue) with the coordinating residues (A22, N27, N286 and T254 labelled and shown as light blue sticks)) and L-Phe in LeuT_W8A_ (light blue) superimposed with Na1 (pink) and the corresponding residues and plus L-Leu in the outward-facing occluded state (PDB-ID: 2A65; 5, light pink)). Right panel shows Na2 (blue) in LeuT_W8A_ (light blue) with the coordinating residues (G20, V23, A351, T354 and S355 labelled and shown as light blue sticks) superimposed with Na2 (pink) and the corresponding residues in the outward-facing occluded state (PDB-ID: 2A65, light pink).

**Supplementary Figure 6.**

**
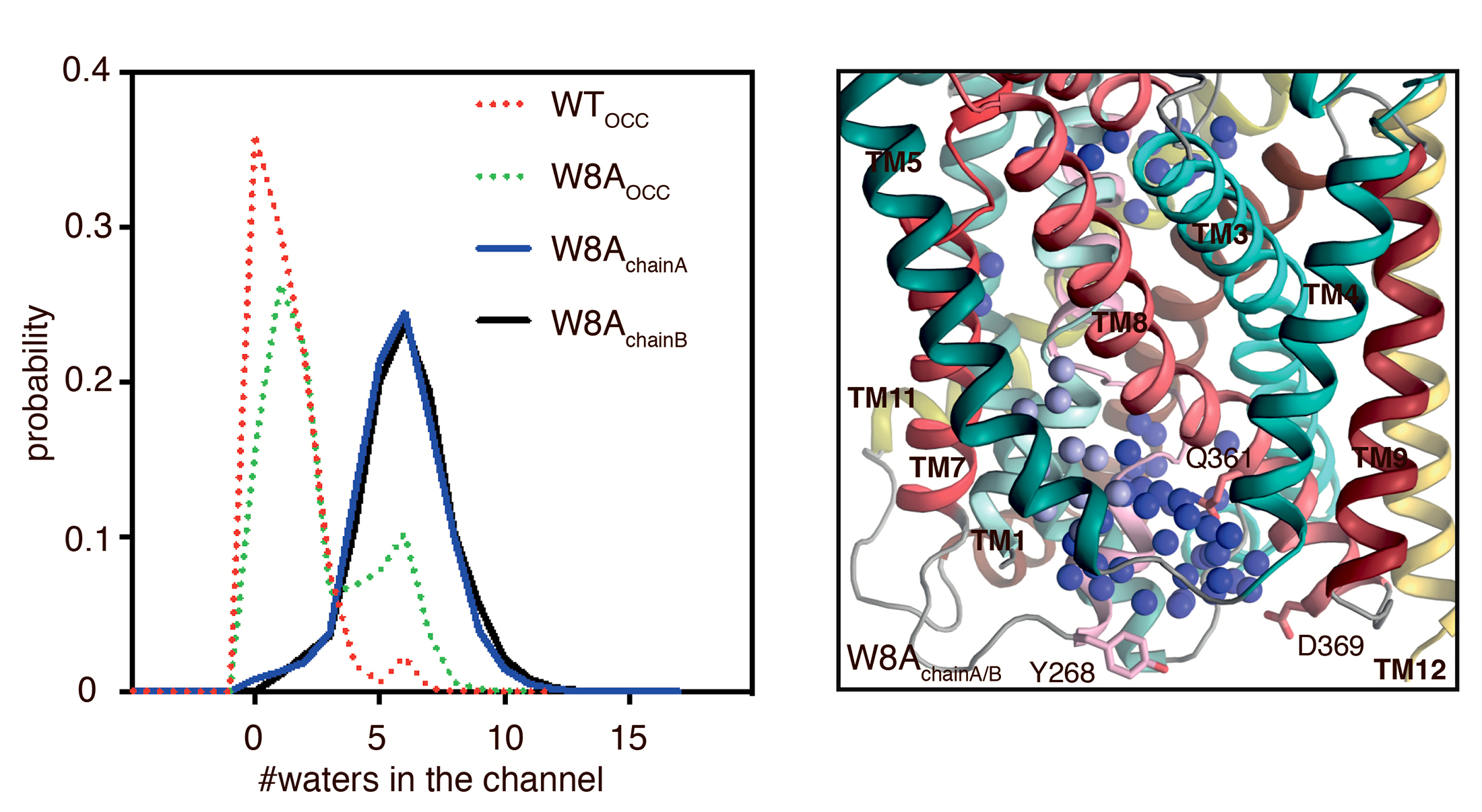
**

a

***
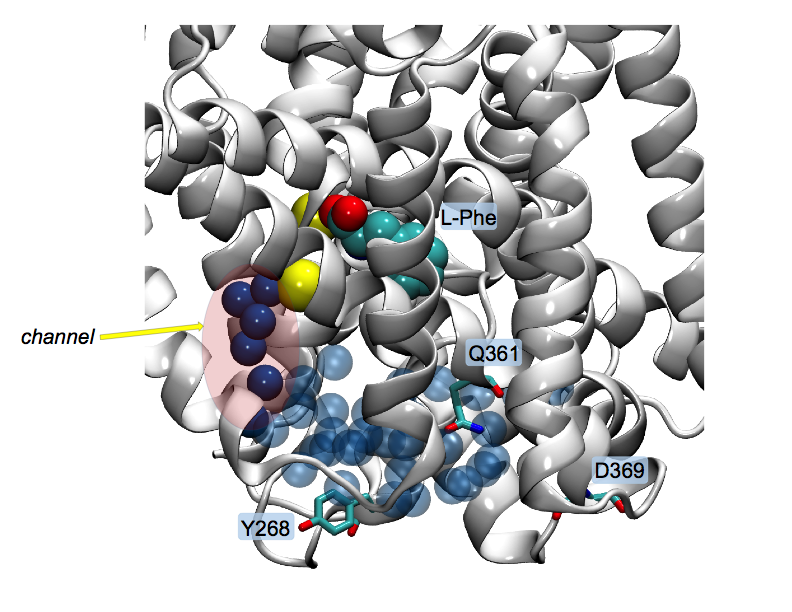
***

b

## **Supplementary Figure 6. Water penetration to the Na^+^/Na2 ion in LeuT_W8A_ crystal structure chains.** (**a**) Histograms of the water counts in the channel region of the intracellular vestibule obtained from analysis of the ensemble MD simulations of crystal structure W8A_chainA_ (blue line) and W8A_chainB_ (black line) LeuT constructs. Counts for WT_OCC_ and W8A_OCC_ constructs (from Supplementary Figure 3) are shown in dashed lines for comparison. **b** Structural representation of the W8A_OUT-OCC_ LeuT highlighting the location of the intracellular channel (semi-transparent red oval). The protein is shown in white cartoon, the residues constituting the internal gate (Y268, D369 and Q361) in licorice and labeled, the two bound Na^+^ ions in yellow spheres and labeled, bound substrate (L-Phe) in space fill and labeled, and oxygen atoms of the water molecules in the intracellular vestibule are depicted as blue spheres (the waters in the channel and outside it are shown in opaque and transparent representations, respectively).

##

**Supplementary Figure 7.**


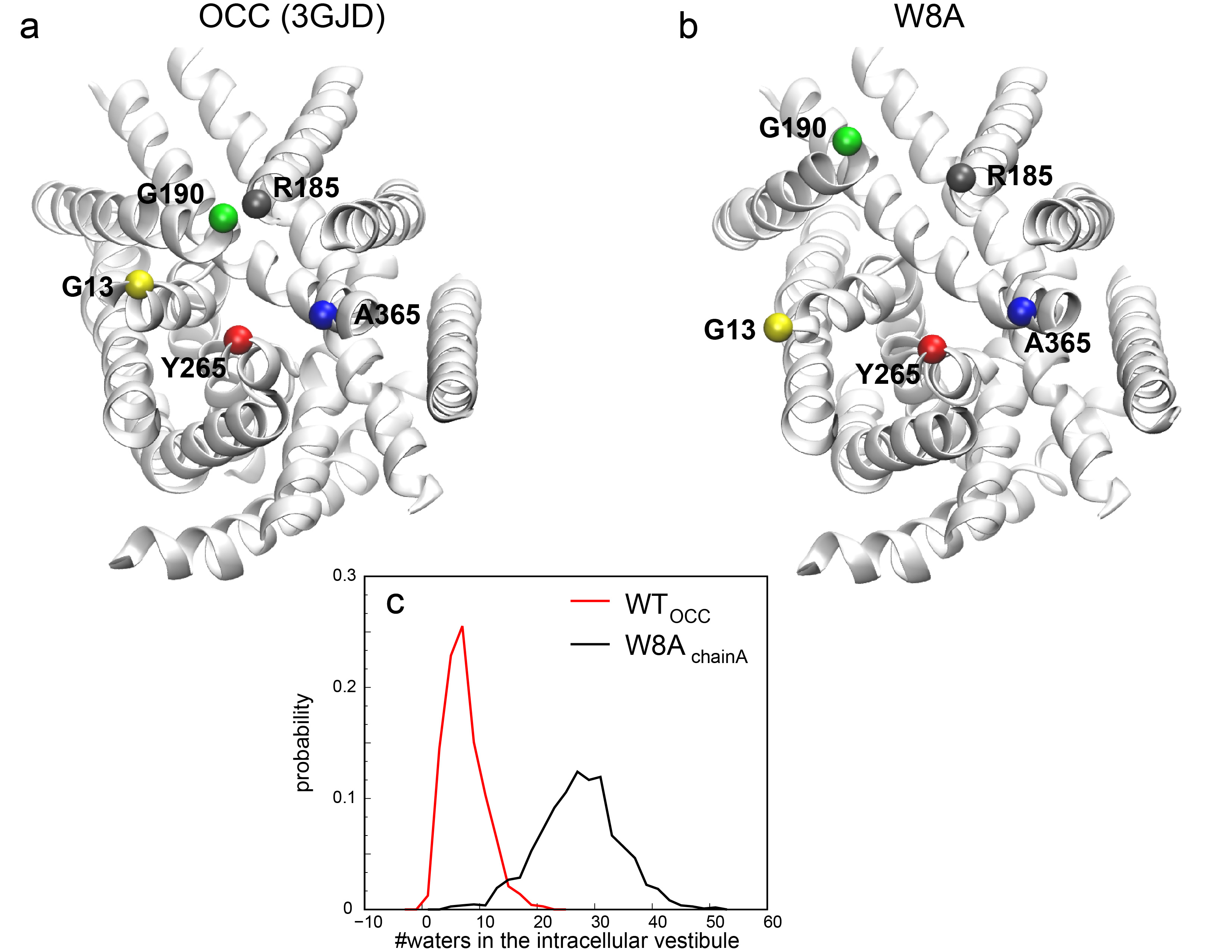


## **Supplementary Figure 7. Calculation of the number of water molecules in the intracellular vestibule in LeuT_W8A_ as compared to LeuT in the outward-facing occluded state. a-b** Views from the intracellular side of LeuT in the outward-facing occluded state (PDBID 3GJD) (**a**) and of the present W8A structure (**b**). The C_α_ atoms of residues G13 (TM1a), R185 (IL2), G190 (IL2), Y265 (TM6b), and A365 (TM8) are shown as spheres of different colors to illustrate differences in the relative positioning of the respective structural segments in the two conformations. **c** Histograms of the water counts in the intracellular vestibule obtained from analysis of the ensemble MD simulations of the WT_OUT-OCC_ (red line) and W8A_OUT-OCC_ (black line) constructs of LeuT.

**Supplementary Figure 8.**

**
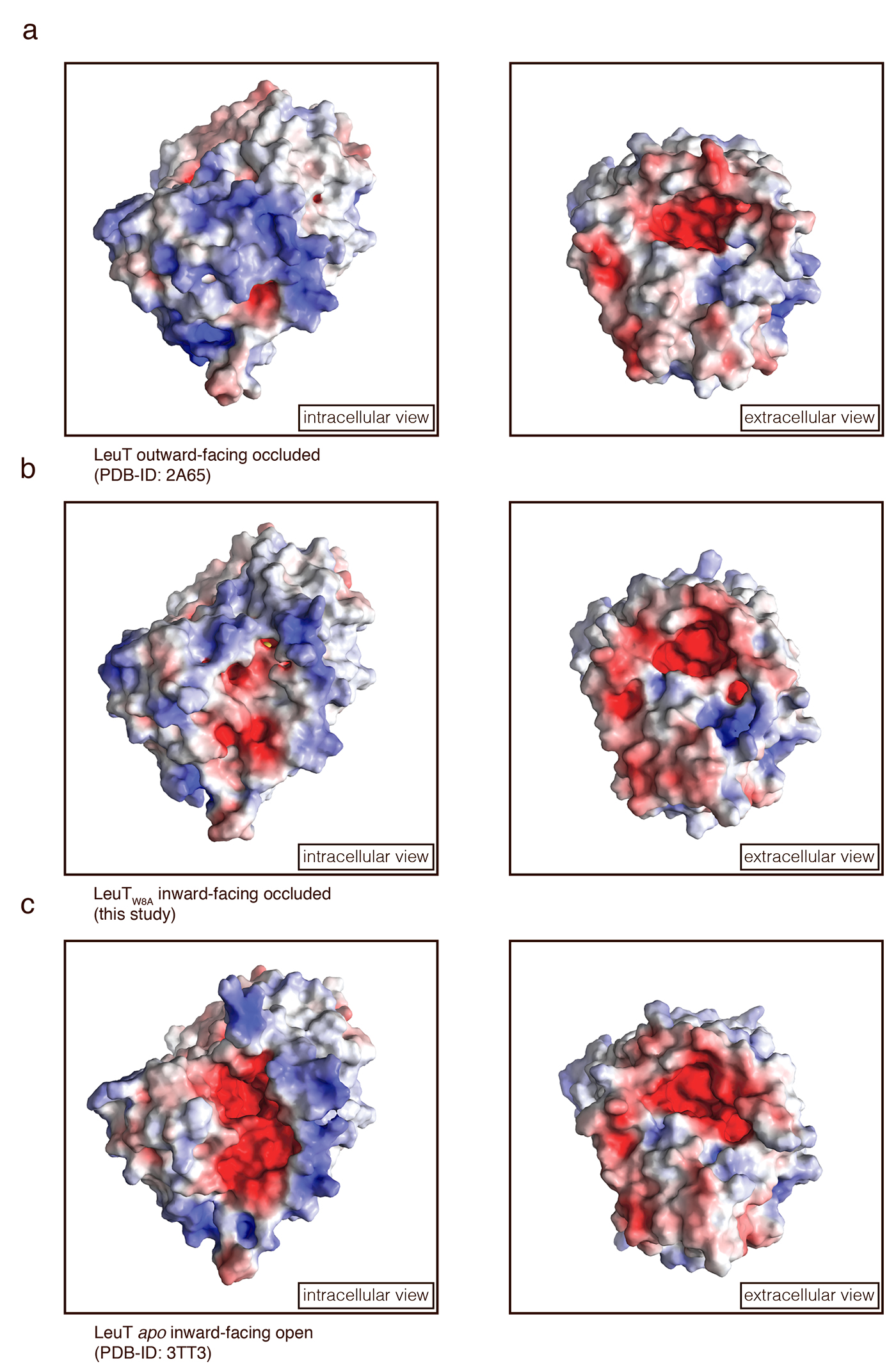
**

## **Supplementary Figure 8. Electrostatic surface potential of intracellular and extracellular vestibules in LeuT_W8A_ and LeuT structures representing preceding and succeeding intermediates of the transport cycle.** Views of intracellular (left panels) and extracellular vestibules (right panels) in **a** LeuT structure in outward-facing occluded state (PDB-ID: 2A65), **b** LeuT_W8A_ structure in inward-facing occluded conformation (this study) and **c** LeuT structure in apo inward-facing open state (PDB-ID: 3TT3). Red and blue colors indicate negative potential and positive potential surfaces, respectively.
