# Supplementary material for: X-ray structure of LeuT in an inward-facing occluded conformation reveals mechanism of substrate release
Source: Nat Commun. 2020 Feb 21;11:1005. doi: 10.1038/s41467-020-14735-w (PMC7035281; doi:10.1038/s41467-020-14735-w)
Supplement: Supplementary file 8 — Description of Additional Supplementary Files [file 41467_2020_14735_MOESM8_ESM.pdf]

Title: Supplementary Movie 1. Side view 1 of morphing between LeuT structures.
Description: The morphing shows the transitions from the outward-facing open (PDB-ID: 3TT1) *via* the outward-facing occluded (PDB-ID: 2A65), the inward-facing occluded (this study) and the *apo* inward-facing open (PDB-ID: 3TT3) to the Na^+/substrate-free outward-facing occluded return (PDB-ID: 5JAE) states, illustrating the overall molecular transitions in LeuT during the transport cycle and in particular movement of TM1a and TM5, and extension of the loop between TM4 and TM5 as LeuT transits from the outward-facing open to the *apo* inward-facing open states. Coloring: TM1 (palecyan), TM5 (deepteal), ECL2 (teal), ECL3 (deep teal), ECL4A (tv red), ECL4B (deepsalmon).

Title: Supplementary Movie 2. Side view 2 of morphing between LeuT structures.
Description: The morphing shows the transitions from the outward-facing open (PDB-ID: 3TT1) *via* the outward-facing occluded (PDB-ID: 2A65), the inward-facing occluded (this study) and the *apo* inward-facing open (PDB-ID: 3TT3) to the Na^+/substrate-free outward-facing occluded return (PDB-ID: 5JAE) states, illustrating the overall molecular transitions in LeuT during the transport cycle and in particular movement of TM1a and TM5, and extension of the loop between TM4 and TM5 as LeuT transits from the outward-facing open to the *apo* inward-facing open states. Coloring: TM1 (palecyan), TM5 (deepteal), ECL2 (teal), ECL3 (deep teal), ECL4A (tv red), ECL4B (deepsalmon).

Title: Supplementary Movie 3. Extracellular view of morphing between LeuT structures.
Description: The morphing shows the transitions from the outward-facing open (PDB-ID: 3TT1) *via* the outward-facing occluded (PDB-ID: 2A65), the inward-facing occluded (this study) and the *apo* inward-facing open (PDB-ID: 3TT3) to the Na^+/substrate-free outward-facing occluded return (PDB-ID: 5JAE) states, illustrating the overall molecular transitions in LeuT during the transport cycle and in particular the closure of the extracellular vestibule as LeuT transits from the outward-facing open to the *apo* inward-facing open states. Coloring: TM1 (palecyan), TM5 (deepteal), ECL2 (teal), ECL3 (deep teal), ECL4A (tv red), ECL4B (deepsalmon).

Title: Supplementary Movie 4. Intracellular view of morphing between LeuT structures.
Description: The morphing shows the transitions from the outward-facing open (PDB-ID: 3TT1) *via* the outward-facing occluded (PDB-ID: 2A65), the inward-facing occluded (this study) and the *apo* inward-facing open (PDB-ID: 3TT3) to the Na^+/substrate-free outward-facing occluded return (PDB-ID: 5JAE) states, illustrating the overall molecular transitions in LeuT during the transport cycle and in particular the opening of a water permeation pathway as LeuT transits from the outward-facing open to the *apo* inward-facing open states. Coloring: TM1 (palecyan), TM5 (deepteal), ECL2 (teal), ECL3 (deep teal), ECL4A (tv red), ECL4B (deepsalmon).
